# 2-(Tritylsulfan­yl)ethyl 2-iodo­benzoate

**DOI:** 10.1107/S1600536811034180

**Published:** 2011-08-27

**Authors:** Xin Zhu, Ping Lu, Seik Weng Ng

**Affiliations:** a Henan University of Traditional Chinese Medicine, Zhengzhou 450008, People’s Republic of China; b Department of Chemistry, University of Malaya, 50603 Kuala Lumpur, Malaysia; c Chemistry Department, King Abdulaziz University, PO Box 80203 Jeddah, Saudi Arabia

## Abstract

The methine C atom of the triphenyl­methyl group in the title compound, C_28_H_23_IO_2_S, is slightly flattened out [ΣC_phen­yl_—C—C_phen­yl_ = 335.6 (5)°]. The –C—O—C—C—S– chain connecting the triphenyl­methyl group and the aromatic ring adopts an extended zigzag conformation, these five atoms lying on an approximate plane (r.m.s. deviation = 0.120 Å).

## Related literature

For the copper(I)-catalysed cleavage of *S*-tritylmethyl thio­ethers, see: Ma *et al.* (2007 ▶); Zhang *et al.* (2009 ▶).
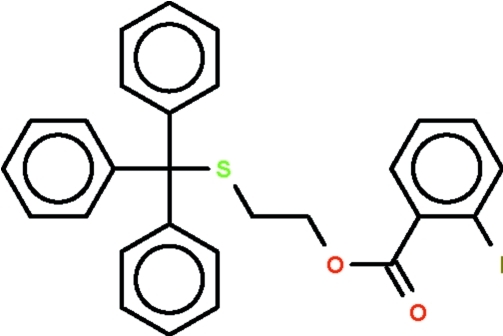


## Experimental

### 

#### Crystal data

C_28_H_23_IO_2_S
*M* *_r_* = 550.42
Monoclinic, 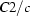

*a* = 28.4378 (4) Å
*b* = 9.6154 (1) Å
*c* = 18.3808 (2) Å
β = 106.618 (1)°
*V* = 4816.13 (10) Å^3^
*Z* = 8
Mo *K*α radiation
μ = 1.44 mm^−1^
*T* = 293 K
0.30 × 0.20 × 0.10 mm

#### Data collection

Bruker SMART APEX diffractometer
Absorption correction: multi-scan (*SADABS*; Sheldrick, 1996 ▶) *T*_min_ = 0.672, *T*_max_ = 0.870
15633 measured reflections
5564 independent reflections
4506 reflections with *I* > 2σ(*I*)
*R*_int_ = 0.017

#### Refinement

*R*[*F* ^2^ > 2σ(*F* ^2^)] = 0.032
*wR*(*F* ^2^) = 0.096
*S* = 1.01
5564 reflections
289 parameters
H-atom parameters constrained
Δρ_max_ = 0.94 e Å^−3^
Δρ_min_ = −0.34 e Å^−3^

### 

Data collection: *APEX2* (Bruker, 2007 ▶); cell refinement: *SAINT* (Bruker, 2007 ▶); data reduction: *SAINT*; program(s) used to solve structure: *SHELXS97* (Sheldrick, 2008 ▶); program(s) used to refine structure: *SHELXL97* (Sheldrick, 2008 ▶); molecular graphics: *X-SEED* (Barbour, 2001 ▶); software used to prepare material for publication: *publCIF* (Westrip, 2010 ▶).

## Supplementary Material

Crystal structure: contains datablock(s) global, I. DOI: 10.1107/S1600536811034180/xu5302sup1.cif

Structure factors: contains datablock(s) I. DOI: 10.1107/S1600536811034180/xu5302Isup2.hkl

Supplementary material file. DOI: 10.1107/S1600536811034180/xu5302Isup3.cml

Additional supplementary materials:  crystallographic information; 3D view; checkCIF report

## Figures and Tables

**Fig. 1.**
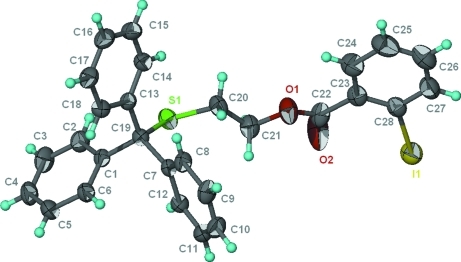
Thermal ellipsoid plot (Barbour, 2001) of C28H23IO2S at the 50% probability level; hydrogen atoms are drawn as spheres of arbitrary radius.

## supplementary crystallographic information

### Comment

Triphenylmethyl is an important *S*-protecting group that prevents a thiol group from reacting with sensitive functional groups. The compound C_28_H_23_IO_2_S (Scheme I) was synthesized for the purpose of examining cupper(I) chloride-catalyzed cleavage investigation (Ma *et al.*, 2007; Zhang *et al.*, 2009). The methine carbon slightly flattened out (ΣC_phenyl_–*C*–C_phenyl_ 335.6 (5) °) owing to decreased crowding by the S atom. The –C–O–C–C–S– chain connecting the triphenylmethyl group and the aromatic ring adopts an extended zigzag conformation, these five atoms lying on an approximate plane (r.m.s. deviation 0.120 Å) (Fig. 1).

### Experimental

A solution of 2-iodobenzoic acid (1.24 g, 5 mmol), dicyclohexylcarbodiimide (1.65 g, 8 mmol) and 4-dimethylaminopyridine (0.98 g, 8 mmol) in THF (20 ml) was stirred for an hour. 2-(Tritylthio)ethanol (1.60 g, 5 mmol) was added. The reaction was stirred for 48 h. The compound was purified by column chromatography with petroleumether–chloroform (3:1) as the eluent. The compound was isolated upon evaporation of the solvent as yellow crystals (2.02 g, 70% yield).

### Refinement

Carbon-bound H-atoms were placed in calculated positions (C—H 0.93 to 0.97 Å) and were included in the refinement in the riding model approximation, with *U*(H) set to 1.2*U*(C).

### Crystal data

**Table:**

C_28_H_23_IO_2_S	*F*(000) = 2208
*M**_r_* = 550.42	*D*_x_ = 1.518 Mg m^−^^3^
Monoclinic, *C*2/*c*	Mo *K*α radiation, λ = 0.71073 Å
Hall symbol: -C 2yc	Cell parameters from 6921 reflections
*a* = 28.4378 (4) Å	θ = 2.4–27.4°
*b* = 9.6154 (1) Å	µ = 1.44 mm^−^^1^
*c* = 18.3808 (2) Å	*T* = 293 K
β = 106.618 (1)°	Prism, yellow
*V* = 4816.13 (10) Å^3^	0.30 × 0.20 × 0.10 mm
*Z* = 8	

### Data collection

**Table:**

Bruker SMART APEX diffractometer	5564 independent reflections
Radiation source: fine-focus sealed tube	4506 reflections with *I* > 2σ(*I*)
graphite	*R*_int_ = 0.017
ω scans	θ_max_ = 27.7°, θ_min_ = 2.3°
Absorption correction: multi-scan (*SADABS*; Sheldrick, 1996)	*h* = −37→33
*T*_min_ = 0.672, *T*_max_ = 0.870	*k* = −11→12
15633 measured reflections	*l* = −23→23

### Refinement

**Table:**

Refinement on *F*^2^	Primary atom site location: structure-invariant direct methods
Least-squares matrix: full	Secondary atom site location: difference Fourier map
*R*[*F*^2^ > 2σ(*F*^2^)] = 0.032	Hydrogen site location: inferred from neighbouring sites
*wR*(*F*^2^) = 0.096	H-atom parameters constrained
*S* = 1.01	*w* = 1/[σ^2^(*F*_o_^2^) + (0.0525*P*)^2^ + 3.6231*P*] where *P* = (*F*_o_^2^ + 2*F*_c_^2^)/3
5564 reflections	(Δ/σ)_max_ = 0.001
289 parameters	Δρ_max_ = 0.94 e Å^−^^3^
0 restraints	Δρ_min_ = −0.34 e Å^−^^3^

### Fractional atomic coordinates and isotropic or equivalent isotropic displacement parameters (Å^2^)

**Table:**

	*x*	*y*	*z*	*U*_iso_*/*U*_eq_	
I1	0.613888 (7)	0.54548 (2)	0.524788 (11)	0.06653 (9)	
S1	0.42074 (2)	1.00910 (7)	0.68754 (4)	0.04999 (15)	
O1	0.48562 (7)	0.6866 (2)	0.62115 (13)	0.0667 (5)	
O2	0.56063 (9)	0.7361 (3)	0.6179 (2)	0.1131 (11)	
C1	0.34622 (8)	1.1936 (2)	0.64315 (12)	0.0395 (4)	
C2	0.36875 (10)	1.2693 (3)	0.70807 (14)	0.0538 (6)	
H2	0.3901	1.2249	0.7494	0.065*	
C3	0.35972 (11)	1.4105 (3)	0.71197 (17)	0.0653 (7)	
H3	0.3759	1.4599	0.7554	0.078*	
C4	0.32767 (12)	1.4781 (3)	0.65340 (18)	0.0632 (7)	
H4	0.3219	1.5727	0.6566	0.076*	
C5	0.30396 (11)	1.4042 (3)	0.58932 (16)	0.0581 (6)	
H5	0.2814	1.4488	0.5494	0.070*	
C6	0.31346 (9)	1.2644 (3)	0.58382 (13)	0.0477 (5)	
H6	0.2977	1.2165	0.5396	0.057*	
C7	0.34474 (8)	0.9869 (2)	0.55660 (12)	0.0378 (4)	
C8	0.31287 (9)	0.8805 (3)	0.52546 (13)	0.0477 (5)	
H8	0.2971	0.8321	0.5556	0.057*	
C9	0.30400 (10)	0.8445 (3)	0.44904 (14)	0.0583 (6)	
H9	0.2826	0.7721	0.4288	0.070*	
C10	0.32654 (11)	0.9150 (3)	0.40389 (14)	0.0607 (7)	
H10	0.3197	0.8927	0.3527	0.073*	
C11	0.35946 (11)	1.0193 (3)	0.43456 (15)	0.0557 (6)	
H11	0.3754	1.0662	0.4042	0.067*	
C12	0.36889 (9)	1.0546 (2)	0.51009 (13)	0.0466 (5)	
H12	0.3916	1.1242	0.5304	0.056*	
C13	0.32141 (8)	0.9602 (2)	0.68042 (12)	0.0394 (4)	
C14	0.33881 (9)	0.8611 (3)	0.73632 (13)	0.0485 (5)	
H14	0.3721	0.8396	0.7514	0.058*	
C15	0.30749 (11)	0.7933 (3)	0.77023 (14)	0.0563 (6)	
H15	0.3199	0.7259	0.8070	0.068*	
C16	0.25806 (10)	0.8250 (3)	0.74968 (14)	0.0562 (6)	
H16	0.2371	0.7797	0.7724	0.067*	
C17	0.24038 (10)	0.9249 (3)	0.69503 (15)	0.0532 (6)	
H17	0.2072	0.9480	0.6812	0.064*	
C18	0.27160 (9)	0.9912 (3)	0.66036 (13)	0.0467 (5)	
H18	0.2590	1.0576	0.6231	0.056*	
C19	0.35420 (8)	1.0362 (2)	0.63906 (12)	0.0385 (4)	
C20	0.43229 (9)	0.8328 (3)	0.66178 (16)	0.0551 (6)	
H20A	0.4385	0.7730	0.7060	0.066*	
H20B	0.4037	0.7976	0.6236	0.066*	
C21	0.47580 (10)	0.8317 (3)	0.63140 (17)	0.0601 (6)	
H21A	0.4686	0.8811	0.5835	0.072*	
H21B	0.5038	0.8752	0.6670	0.072*	
C22	0.52920 (9)	0.6522 (3)	0.61484 (16)	0.0581 (6)	
C23	0.53339 (9)	0.4982 (3)	0.60730 (14)	0.0515 (6)	
C24	0.50377 (11)	0.4115 (4)	0.63571 (18)	0.0675 (7)	
H24	0.4814	0.4507	0.6580	0.081*	
C25	0.50676 (12)	0.2688 (4)	0.6317 (2)	0.0801 (9)	
H25	0.4869	0.2125	0.6516	0.096*	
C26	0.53938 (13)	0.2107 (4)	0.5980 (2)	0.0790 (9)	
H26	0.5415	0.1145	0.5950	0.095*	
C27	0.56871 (11)	0.2933 (3)	0.56890 (16)	0.0652 (7)	
H27	0.5904	0.2527	0.5457	0.078*	
C28	0.56646 (9)	0.4366 (3)	0.57364 (14)	0.0506 (6)	

### Atomic displacement parameters (Å^2^)

**Table:**

	*U*^11^	*U*^22^	*U*^33^	*U*^12^	*U*^13^	*U*^23^
I1	0.05710 (13)	0.06887 (15)	0.07605 (15)	0.00487 (8)	0.02295 (9)	0.00746 (9)
S1	0.0392 (3)	0.0480 (3)	0.0585 (3)	0.0024 (2)	0.0071 (2)	0.0001 (3)
O1	0.0485 (10)	0.0558 (11)	0.0997 (14)	0.0031 (9)	0.0275 (9)	−0.0087 (11)
O2	0.0711 (14)	0.0704 (16)	0.215 (3)	−0.0192 (13)	0.0685 (18)	−0.0479 (19)
C1	0.0402 (10)	0.0371 (11)	0.0428 (10)	−0.0014 (9)	0.0145 (8)	−0.0039 (9)
C2	0.0558 (14)	0.0530 (15)	0.0490 (12)	0.0005 (11)	0.0094 (10)	−0.0101 (11)
C3	0.0713 (17)	0.0556 (16)	0.0688 (16)	−0.0108 (14)	0.0200 (14)	−0.0277 (14)
C4	0.0789 (19)	0.0374 (14)	0.0806 (19)	0.0029 (13)	0.0346 (16)	−0.0084 (13)
C5	0.0715 (17)	0.0439 (14)	0.0613 (14)	0.0122 (13)	0.0226 (13)	0.0062 (12)
C6	0.0531 (13)	0.0414 (13)	0.0472 (11)	0.0047 (10)	0.0122 (10)	−0.0028 (10)
C7	0.0406 (10)	0.0333 (10)	0.0404 (10)	0.0039 (8)	0.0128 (8)	−0.0014 (8)
C8	0.0500 (12)	0.0442 (13)	0.0520 (12)	−0.0025 (10)	0.0197 (10)	−0.0074 (10)
C9	0.0576 (14)	0.0584 (16)	0.0587 (14)	−0.0045 (12)	0.0161 (11)	−0.0207 (13)
C10	0.0691 (16)	0.0694 (18)	0.0443 (12)	0.0090 (14)	0.0175 (11)	−0.0094 (12)
C11	0.0704 (16)	0.0532 (15)	0.0497 (13)	0.0091 (13)	0.0269 (12)	0.0060 (11)
C12	0.0542 (13)	0.0389 (12)	0.0489 (12)	0.0010 (10)	0.0184 (10)	0.0017 (10)
C13	0.0457 (11)	0.0358 (11)	0.0376 (10)	−0.0016 (9)	0.0133 (8)	−0.0049 (8)
C14	0.0562 (13)	0.0424 (13)	0.0471 (11)	0.0038 (11)	0.0152 (10)	0.0015 (10)
C15	0.0778 (17)	0.0461 (14)	0.0486 (12)	−0.0006 (13)	0.0238 (12)	0.0049 (11)
C16	0.0701 (16)	0.0510 (15)	0.0561 (13)	−0.0126 (13)	0.0315 (12)	−0.0054 (12)
C17	0.0498 (13)	0.0575 (15)	0.0570 (13)	−0.0053 (11)	0.0228 (11)	−0.0069 (12)
C18	0.0483 (12)	0.0465 (13)	0.0456 (11)	0.0010 (10)	0.0141 (10)	0.0020 (10)
C19	0.0377 (10)	0.0362 (11)	0.0406 (10)	0.0010 (8)	0.0098 (8)	0.0012 (8)
C20	0.0473 (13)	0.0479 (14)	0.0692 (15)	0.0100 (11)	0.0154 (11)	0.0047 (12)
C21	0.0527 (14)	0.0559 (16)	0.0718 (16)	0.0020 (12)	0.0181 (12)	−0.0063 (13)
C22	0.0429 (12)	0.0636 (17)	0.0682 (15)	−0.0021 (12)	0.0164 (11)	−0.0129 (13)
C23	0.0399 (12)	0.0550 (14)	0.0537 (13)	0.0027 (11)	0.0038 (10)	−0.0012 (12)
C24	0.0514 (14)	0.0707 (19)	0.0801 (19)	0.0011 (14)	0.0182 (13)	0.0079 (16)
C25	0.0674 (19)	0.072 (2)	0.098 (2)	−0.0073 (17)	0.0195 (17)	0.0247 (19)
C26	0.083 (2)	0.0524 (18)	0.095 (2)	0.0014 (16)	0.0163 (18)	0.0085 (17)
C27	0.0673 (17)	0.0558 (17)	0.0693 (16)	0.0127 (14)	0.0146 (13)	0.0021 (14)
C28	0.0438 (12)	0.0512 (14)	0.0512 (12)	0.0043 (10)	0.0048 (10)	0.0019 (11)

### Geometric parameters (Å, °)

**Table:**

I1—C28	2.101 (3)	C12—H12	0.9300
S1—C20	1.815 (3)	C13—C14	1.385 (3)
S1—C19	1.865 (2)	C13—C18	1.390 (3)
O1—C22	1.319 (3)	C13—C19	1.544 (3)
O1—C21	1.445 (4)	C14—C15	1.387 (4)
O2—C22	1.193 (4)	C14—H14	0.9300
C1—C2	1.388 (3)	C15—C16	1.381 (4)
C1—C6	1.392 (3)	C15—H15	0.9300
C1—C19	1.536 (3)	C16—C17	1.376 (4)
C2—C3	1.387 (4)	C16—H16	0.9300
C2—H2	0.9300	C17—C18	1.388 (4)
C3—C4	1.360 (5)	C17—H17	0.9300
C3—H3	0.9300	C18—H18	0.9300
C4—C5	1.375 (4)	C20—C21	1.496 (4)
C4—H4	0.9300	C20—H20A	0.9700
C5—C6	1.381 (4)	C20—H20B	0.9700
C5—H5	0.9300	C21—H21A	0.9700
C6—H6	0.9300	C21—H21B	0.9700
C7—C8	1.378 (3)	C22—C23	1.495 (4)
C7—C12	1.401 (3)	C23—C24	1.389 (4)
C7—C19	1.537 (3)	C23—C28	1.397 (4)
C8—C9	1.398 (3)	C24—C25	1.378 (5)
C8—H8	0.9300	C24—H24	0.9300
C9—C10	1.365 (4)	C25—C26	1.373 (5)
C9—H9	0.9300	C25—H25	0.9300
C10—C11	1.378 (4)	C26—C27	1.366 (5)
C10—H10	0.9300	C26—H26	0.9300
C11—C12	1.379 (3)	C27—C28	1.383 (4)
C11—H11	0.9300	C27—H27	0.9300
			
C20—S1—C19	103.90 (11)	C17—C16—H16	120.5
C22—O1—C21	118.3 (2)	C15—C16—H16	120.5
C2—C1—C6	117.3 (2)	C16—C17—C18	120.5 (2)
C2—C1—C19	121.4 (2)	C16—C17—H17	119.7
C6—C1—C19	121.23 (19)	C18—C17—H17	119.7
C3—C2—C1	120.7 (2)	C17—C18—C13	121.1 (2)
C3—C2—H2	119.6	C17—C18—H18	119.4
C1—C2—H2	119.6	C13—C18—H18	119.4
C4—C3—C2	121.2 (3)	C1—C19—C7	111.43 (17)
C4—C3—H3	119.4	C1—C19—C13	108.88 (17)
C2—C3—H3	119.4	C7—C19—C13	112.31 (17)
C3—C4—C5	119.0 (3)	C1—C19—S1	104.96 (14)
C3—C4—H4	120.5	C7—C19—S1	107.20 (14)
C5—C4—H4	120.5	C13—C19—S1	111.83 (14)
C4—C5—C6	120.5 (3)	C21—C20—S1	109.6 (2)
C4—C5—H5	119.8	C21—C20—H20A	109.8
C6—C5—H5	119.8	S1—C20—H20A	109.8
C5—C6—C1	121.3 (2)	C21—C20—H20B	109.8
C5—C6—H6	119.3	S1—C20—H20B	109.8
C1—C6—H6	119.3	H20A—C20—H20B	108.2
C8—C7—C12	118.1 (2)	O1—C21—C20	105.5 (2)
C8—C7—C19	123.2 (2)	O1—C21—H21A	110.6
C12—C7—C19	118.7 (2)	C20—C21—H21A	110.6
C7—C8—C9	120.6 (2)	O1—C21—H21B	110.6
C7—C8—H8	119.7	C20—C21—H21B	110.6
C9—C8—H8	119.7	H21A—C21—H21B	108.8
C10—C9—C8	120.5 (3)	O2—C22—O1	122.4 (3)
C10—C9—H9	119.8	O2—C22—C23	126.5 (3)
C8—C9—H9	119.8	O1—C22—C23	111.0 (2)
C9—C10—C11	119.7 (2)	C24—C23—C28	118.0 (3)
C9—C10—H10	120.2	C24—C23—C22	119.1 (3)
C11—C10—H10	120.2	C28—C23—C22	122.9 (2)
C10—C11—C12	120.3 (2)	C25—C24—C23	121.7 (3)
C10—C11—H11	119.8	C25—C24—H24	119.2
C12—C11—H11	119.8	C23—C24—H24	119.2
C11—C12—C7	120.8 (2)	C26—C25—C24	119.3 (3)
C11—C12—H12	119.6	C26—C25—H25	120.4
C7—C12—H12	119.6	C24—C25—H25	120.4
C14—C13—C18	117.7 (2)	C27—C26—C25	120.5 (3)
C14—C13—C19	123.5 (2)	C27—C26—H26	119.8
C18—C13—C19	118.74 (19)	C25—C26—H26	119.8
C13—C14—C15	121.2 (2)	C26—C27—C28	120.6 (3)
C13—C14—H14	119.4	C26—C27—H27	119.7
C15—C14—H14	119.4	C28—C27—H27	119.7
C16—C15—C14	120.5 (2)	C27—C28—C23	120.0 (3)
C16—C15—H15	119.8	C27—C28—I1	115.0 (2)
C14—C15—H15	119.8	C23—C28—I1	125.0 (2)
C17—C16—C15	119.0 (2)		
			
C6—C1—C2—C3	−1.9 (4)	C12—C7—C19—C13	174.8 (2)
C19—C1—C2—C3	−177.5 (2)	C8—C7—C19—S1	119.2 (2)
C1—C2—C3—C4	1.8 (5)	C12—C7—C19—S1	−62.0 (2)
C2—C3—C4—C5	0.0 (5)	C14—C13—C19—C1	−128.4 (2)
C3—C4—C5—C6	−1.6 (4)	C18—C13—C19—C1	52.3 (3)
C4—C5—C6—C1	1.5 (4)	C14—C13—C19—C7	107.7 (2)
C2—C1—C6—C5	0.3 (4)	C18—C13—C19—C7	−71.6 (3)
C19—C1—C6—C5	175.9 (2)	C14—C13—C19—S1	−12.9 (3)
C12—C7—C8—C9	−1.9 (4)	C18—C13—C19—S1	167.80 (17)
C19—C7—C8—C9	177.0 (2)	C20—S1—C19—C1	−166.42 (15)
C7—C8—C9—C10	−0.4 (4)	C20—S1—C19—C7	−47.83 (17)
C8—C9—C10—C11	2.1 (4)	C20—S1—C19—C13	75.69 (17)
C9—C10—C11—C12	−1.3 (4)	C19—S1—C20—C21	129.09 (19)
C10—C11—C12—C7	−1.0 (4)	C22—O1—C21—C20	−161.8 (2)
C8—C7—C12—C11	2.6 (3)	S1—C20—C21—O1	173.37 (18)
C19—C7—C12—C11	−176.3 (2)	C21—O1—C22—O2	0.6 (5)
C18—C13—C14—C15	1.1 (3)	C21—O1—C22—C23	178.1 (2)
C19—C13—C14—C15	−178.2 (2)	O2—C22—C23—C24	153.0 (4)
C13—C14—C15—C16	−1.1 (4)	O1—C22—C23—C24	−24.4 (4)
C14—C15—C16—C17	0.1 (4)	O2—C22—C23—C28	−26.1 (5)
C15—C16—C17—C18	0.9 (4)	O1—C22—C23—C28	156.5 (2)
C16—C17—C18—C13	−0.8 (4)	C28—C23—C24—C25	0.5 (4)
C14—C13—C18—C17	−0.2 (3)	C22—C23—C24—C25	−178.7 (3)
C19—C13—C18—C17	179.2 (2)	C23—C24—C25—C26	−0.8 (5)
C2—C1—C19—C7	−156.2 (2)	C24—C25—C26—C27	0.2 (5)
C6—C1—C19—C7	28.3 (3)	C25—C26—C27—C28	0.8 (5)
C2—C1—C19—C13	79.4 (3)	C26—C27—C28—C23	−1.1 (4)
C6—C1—C19—C13	−96.1 (2)	C26—C27—C28—I1	−179.5 (2)
C2—C1—C19—S1	−40.5 (3)	C24—C23—C28—C27	0.5 (4)
C6—C1—C19—S1	144.05 (18)	C22—C23—C28—C27	179.6 (2)
C8—C7—C19—C1	−126.5 (2)	C24—C23—C28—I1	178.69 (19)
C12—C7—C19—C1	52.3 (3)	C22—C23—C28—I1	−2.2 (3)
C8—C7—C19—C13	−4.0 (3)		
